# Simultaneous Hospital Outbreaks of New Delhi Metallo-β-Lactamase-Producing *Enterobacterales* Unraveled Using Whole-Genome Sequencing

**DOI:** 10.1128/spectrum.02287-21

**Published:** 2022-03-21

**Authors:** Stéphane Lo, Isabelle Lolom, Valérie Goldstein, Marie Petitjean, Emilie Rondinaud, Vincent Bunel-Gourdy, Alexy Tran Dinh, Paul-Henri Wicky, Etienne Ruppé, Camille d’Humières, Jean-Christophe Lucet, Laurence Armand-Lefèvre

**Affiliations:** a Bacteriology Department, Bichat Claude Bernard University Hospital, AP-HP Nord-University of Paris, Paris, France; b INSERM, IAME UMR 1137, University of Paris, Paris, France; c Infection Control Unit, Bichat Claude Bernard University Hospital, AP-HP Nord-University of Paris, Paris, France; d Pneumology Department, Bichat Claude Bernard University Hospital, AP-HP Nord-University of Paris, Paris, France; e Surgical ICU, Bichat Claude Bernard University Hospital, AP-HP Nord-University of Paris, Paris, France; f INSERM, UMR 1148, University of Paris, Paris, France; g Medical and Infectious Diseases ICU, Bichat Claude Bernard University Hospital, AP-HP Nord-University of Paris, Paris, France; Universidad de Buenos Aires, Facultad de Farmacia y Bioquímica

**Keywords:** whole-genome sequencing, carbapenemase-producing *Enterobacterales*, outbreak, NDM, *Klebsiella pneumoniae*, *Klebsiella oxytoca*

## Abstract

Multidrug-resistant *Enterobacterales*, including carbapenemase producers, are currently spreading in health care facilities and the community. The Bichat Claude Bernard hospital in Paris faced a prolonged NDM-producing *Enterobacterales* (NDM-CPE) outbreak. Whole-genome sequencing (WGS) was performed on all isolated NDM-CPE to evaluate its benefits for outbreak surveillance and comprehension. All NDM-CPE isolates collected during the outbreak period (August 2016 to January 2018) were sequenced using the Illumina NextSeq platform. Gene content and core genomes were compared. Genomics results underwent epidemiological analysis which classified NDM-CPE cases as imported (positive sample during the 48 h after admission), hospital acquired, or uncertain. Over the epidemic period, 61 patients were colonized or infected with 81 distinct NDM-CPE isolates. Klebsiella pneumoniae was the most common species (*n* = 52, 64%), followed by Escherichia coli (13.5%) and other species (22.5%). In all, 43/52 (83%) K. pneumoniae isolates were clonal (≤18 single nucleotide polymorphisms [SNPs] except for three isolates) and belonged to ST307. The IncFIIK [K2:A-/B-] plasmid carrying *bla*_NDM-1_ present in all ST307 K. pneumoniae isolates was also detected in 18 other NDM-CPE isolates. Additionally, eight clonal ST144 Klebsiella oxytoca (≤18 SNPs) isolates lacking the epidemic plasmid were observed. The WGS analyses confirmed the acquired and imported cases except for two patients and resolved uncertain cases, which all turned out to be hospital acquisitions. WGS coupled with epidemiological analysis unraveled three epidemic phenomena: mainly the spread of a clonal ST307 K. pneumoniae strain and its conjugative plasmid carrying *bla*_NDM-1_ but also the unexpected clonal spread of an ST144 K. oxytoca strain.

**IMPORTANCE** Carbapenemase-producing *Enterobacterales* (CPE) can spread and cause outbreaks in health care facilities, resulting in increased lengths of stay and morbidity. Control of outbreaks requires epidemiological surveillance, usually based on microbiological screening and patient follow-up. These data are sometimes insufficient to identify the routes of dissemination. There is therefore a need for more accurate tools such as whole-genome sequencing (WGS), which allows comparison of isolates but also plasmids carrying resistance with a high definition. In this work, we retrospectively sequenced the genomes of all NDM-producing *Enterobacterales* isolated during a prolonged NDM outbreak in our hospital. We demonstrated the value of combining WGS with epidemiological data that unveiled the multiple mechanisms of dissemination involved in the outbreak and confirmed transmission cases. This work reinforces the potential of WGS in outbreak surveillance and suggests that it could improve outbreak control if used in real time by confirming transmission cases more rapidly.

## INTRODUCTION

Global reports of carbapenemase-producing *Enterobacterales* (CPE) are increasing worldwide (1). CPE represent a major threat for public health when responsible for infection, with high mortality and prolongation of hospital length of stay (2). According to the European Antimicrobial Resistance Surveillance Network (EARS-Net) in 2019, the prevalence of carbapenem resistance in Klebsiella pneumoniae and Escherichia coli ranged from <1% to 58% depending on the country but remained for the majority below 10% (3). With the support of nationally implemented surveillance and infection control measures (4), the proportion of carbapenem resistance among *Enterobacterales* remained at 1% in 2019 in France (3). Most hospital outbreaks in countries with low CPE prevalence can be controlled by enhanced control measures, based on extensive screening of contact patients and strict isolation precautions (4–6). Several large hospital outbreaks have been reported, with successful control in France (5, 7, 8). However, in some cases (9, 10), genomic analysis was required to decipher complex or prolonged outbreaks.

Several carbapenemase enzymes have been described to date, including the New Delhi metallo-β-lactamase-type carbapenemase (NDM), which is disseminated worldwide, mainly in health care facilities (6, 11–13). In Ambler’s structural classification, NDM is a class B enzyme which can hydrolyze all β-lactams except monobactams, and more than 20 variants have been described so far (11). In France, NDM is the second most identified CPE enzyme, accounting for 16% of all carbapenemases, mostly detected in K. pneumoniae species (14).

The Bichat Claude Bernard (BCB) University Hospital, located in Paris (France), faced an outbreak of NDM-producing *Enterobacterales* (NDM-CPE) in multiple wards between 2016 and 2018. The outbreak involved multiple species of *Enterobacterales* from patients not always epidemiologically linked and was difficult to control, leading to the need for accurate molecular typing to clarify the routes of transmission. We therefore performed whole-genome sequencing (WGS) on all NDM-CPE identified in our hospital during the epidemic period, and WGS results then underwent epidemiological analysis. This approach was evaluated for its additional value in outbreak surveillance and characterization of its mechanisms.

## RESULTS

### Outbreak description.

**FIG 1:**
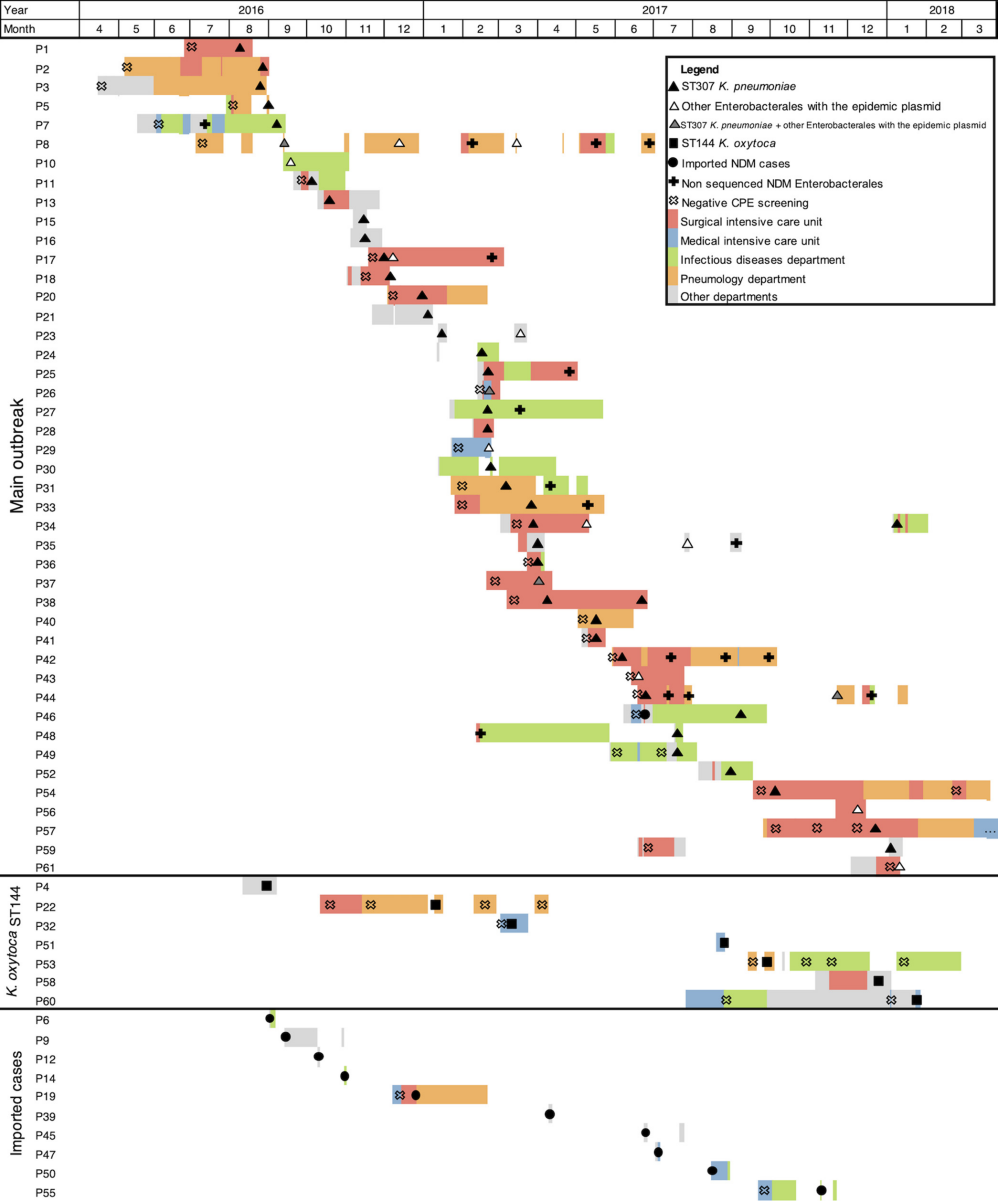
Outbreak timeline displaying the location of all the New Delhi metallo-β-lactamase-producing *Enterobacterales* (NDM-CPE) carriers over time, NDM-CPE isolates, and negative rectal screenings. Patients are identified by their number, P1 to P61. “Other departments” refers to the emergency, endocrinology, cardiology, orthopedic surgery, digestive surgery, gynecology, internal medicine, and otorhinolaryngology departments. Patient P57 stayed at Bichat Hospital until August 2018.

From 1 August 2016 to 31 January 2018, an NDM-CPE-positive culture was detected in 61 patients. Among them, 34 (56%) were men with an average age of 63 years (range, 23 to 94). In all, the outbreak involved 13 different clinical units in which a case stayed at least once, but four units were mainly involved: the surgical intensive care unit (ICU), the medical ICU, the lung transplant/pneumology unit, and the infectious diseases department (Fig. 1). An NDM-CPE isolate was detected within 48 h of hospital admission in 10/61 (16%) patients.

Overall, 38 patients were epidemiologically classified as acquired cases, 13 had an uncertain acquisition, and 10 were classified as imported cases. An index case could not be confirmed. Of the 51 acquired or uncertain cases, 31 were only carriers during their first hospital stay and 20 had a positive clinical culture, including 5 with bloodstream infection (see the Supplementary 1 table in the supplemental material).

The outbreak was protracted due to the very long hospital stays or repeated hospital stays of several case patients. Seven patients stayed for more than 2.5 consecutive months in the same unit, including one lung transplant patient staying for 7 months between the lung transplant unit and the surgical ICU (P54 in Fig. 1). Further, patient P57 acquired an NDM-CPE isolate during the first 3 months of stay and was hospitalized for 11 months between the surgical ICU and the pneumology department. Cohorting with dedicated health care workers (HCW) was organized on five occasions, for a total duration of 414 days. Transmissions from cohorted patients occurred, however, either due to noncompliance with recommended measures, e.g., in emergency ICU situations, or to very long duration of cohorting with HCW fatigue and less attention to cohorting.

### Isolate characterization.

During the outbreak period, 85 nonduplicate NDM-CPE isolates were obtained. Three isolates were not retrieved, and one had a nonanalyzable sequence due to too few reads; thus, 81 isolates were included in the analysis and sequenced. The average number of trimmed reads per genome was 2.7 million, which corresponds to a sequencing depth of 73×.

Isolates belonged to five species: Klebsiella pneumoniae (*n* = 52, 64%), Klebsiella oxytoca (*n* = 11, 13.5%), Escherichia coli (*n* = 11, 13.5%), Enterobacter cloacae (*n* = 5, 6%), and Citrobacter freundii (*n* = 2, 2%) (see the Supplementary 2 figure).

The isolates were mainly retrieved from screening samples: 68 from rectal and four from oropharyngeal screenings. The clinical samples included blood cultures (*n* = 2), urine (*n* = 2), skin swabs (*n* = 2), catheter (*n* = 2), and the respiratory tract sample of a ventilated patient (*n* = 1) (see the Supplementary 1 table).

### Antibiotic susceptibility and resistance genes.

All 81 isolates were phenotypically resistant to penicillins, third- and fourth-generation cephalosporins, and carbapenems. Regarding other antibiotic classes, 73 (90%) isolates were resistant to ciprofloxacin, 25 (31%) to gentamicin, and 16 (20%) to amikacin (Supplementary 3 table).

A total of 70 different resistance genes including 18 β-lactamase genes were identified (Supplementary 4 table). As expected, *bla*_NDM_ was detected in all genomes. The *bla*_NDM-1_ variant was the most frequent (*n* = 76, 94%) followed by *bla*_NDM-5_ (*n* = 4, 3 E. coli and 1 K. pneumoniae) and *bla*_NDM-7_ (*n* = 1 E. cloacae). Isolate EPINDM75, which was detected from an imported case, carried concomitantly a *bla*_NDM-1_ and a *bla*_OXA-48_ gene. Beside carbapenemases genes, *bla*_CTX-M-15_ and *bla*_OXA-9_ were also frequently detected, in 78 (96%) and 60 (66%) isolates, respectively. The plasmid-mediated quinolone resistance gene, *qnrS1*, was found in 73 (90%) isolates.

### Multilocus sequence type (MLST) typing.

**FIG 2:**
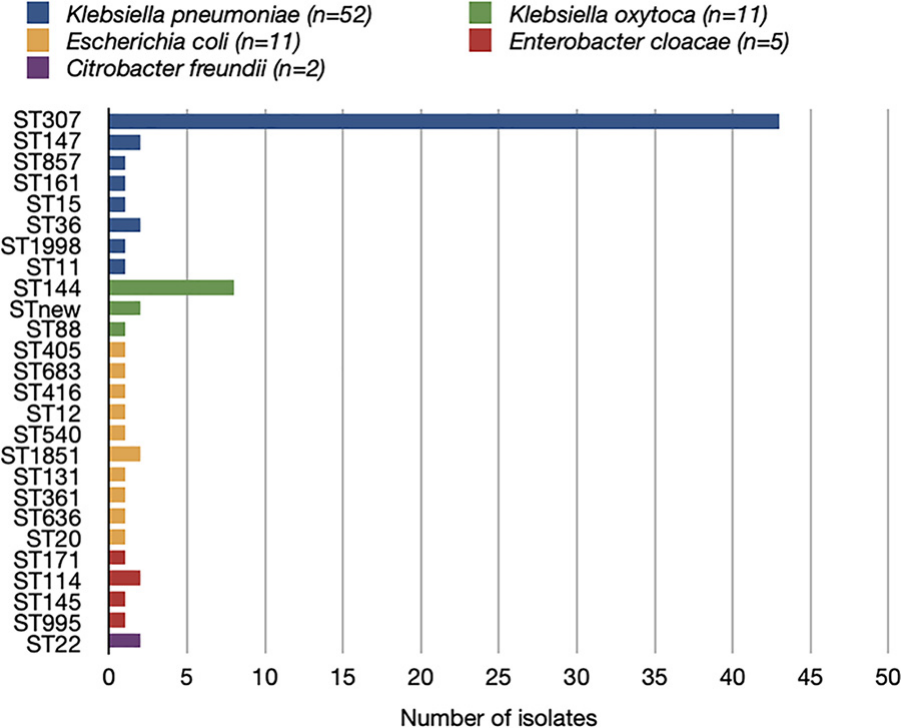
Sequence type distribution by *Enterobacterales* species for the 81 sequenced New Delhi metallo-β-lactamase-producing *Enterobacterales* isolates.

The 52 K. pneumoniae isolates were distributed into eight STs, among which ST307 predominated (*n* = 43, 83%), ST36 and ST147 each contained two isolates, and the remainders were singletons. Concerning other species, 8/11 (73%) K. oxytoca isolates belonged to ST144, 2/5 E. cloacae isolates belonged to ST114, and 2/2 C. freundii isolates belonged to ST22. All remaining isolates were singletons (Fig. 2).

### Virulence genes and polysaccharide capsule typing.

None of the K. pneumoniae or the K. oxytoca genomes carried colibactin, aerobactin, salmochelin, or hypermucoviscosity genes. Yersiniabactin genes were found only in three nonepidemic isolates, EPINDM11, 67, and 75.

**FIG 3:**
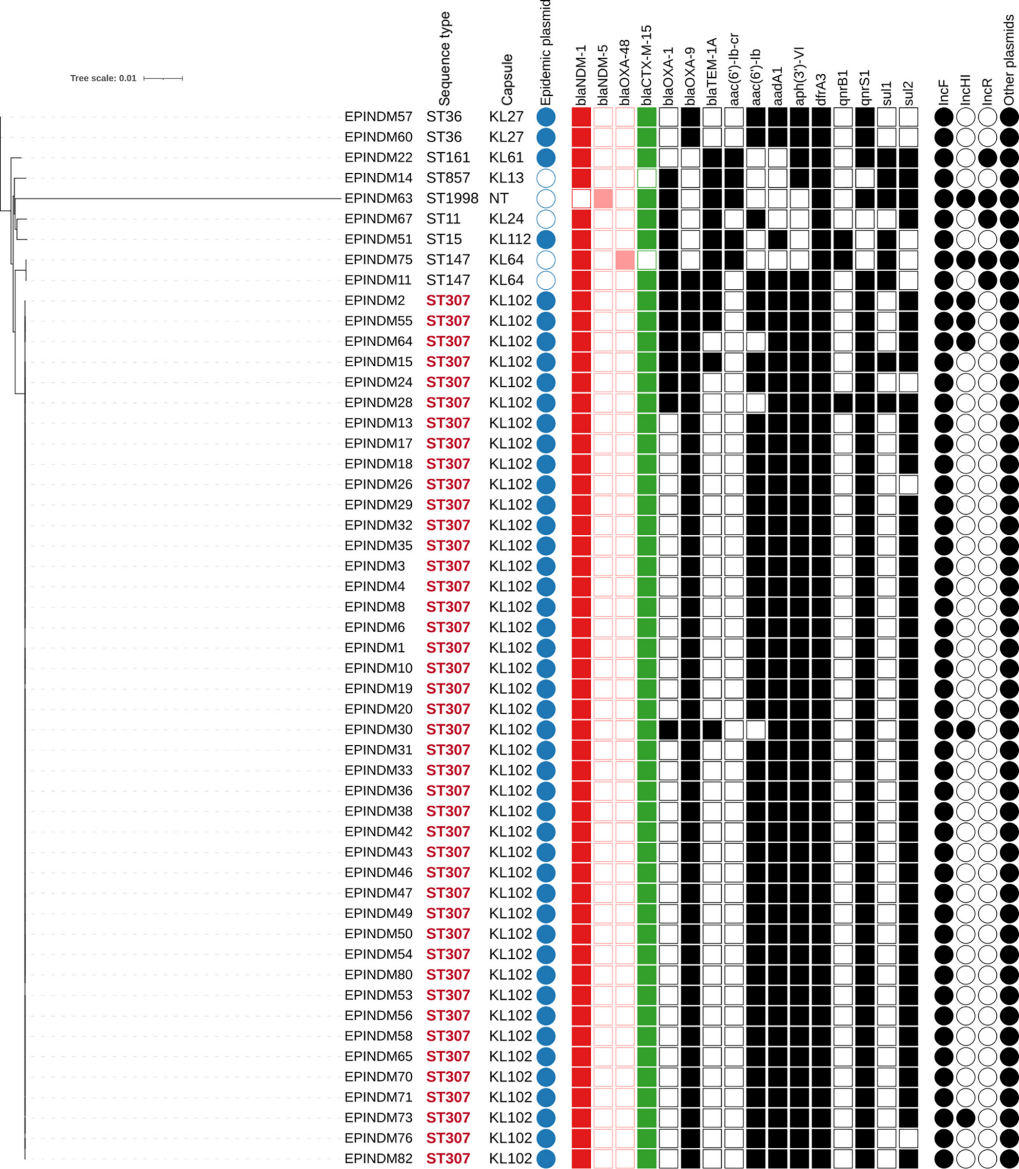
Klebsiella pneumoniae phylogenetic tree based on single nucleotide polymorphisms. Filled shapes indicate the presence of the corresponding genes or plasmids. NT, nontypeable.

Capsule typing of all K. pneumoniae isolates showed that each ST was associated with a capsule type (Fig. 3): ST307 with KL102, ST147 with KL64, ST15 with KL112, ST11 with KL24, ST857 with KL13, ST161 with KL61, and ST36 with KL27.

### Plasmid analysis.

Plasmid replicon typing identified 11 different incompatibility groups (Supplementary 5 table). IncF-type plasmids were highly predominant and detected in 80 (99%) isolates, IncHI plasmids were detected in 18 (22%) isolates, IncR in 6 (7%) isolates, and IncI in 5 (6%) isolates.

### Phylogeny.

**FIG 4:**
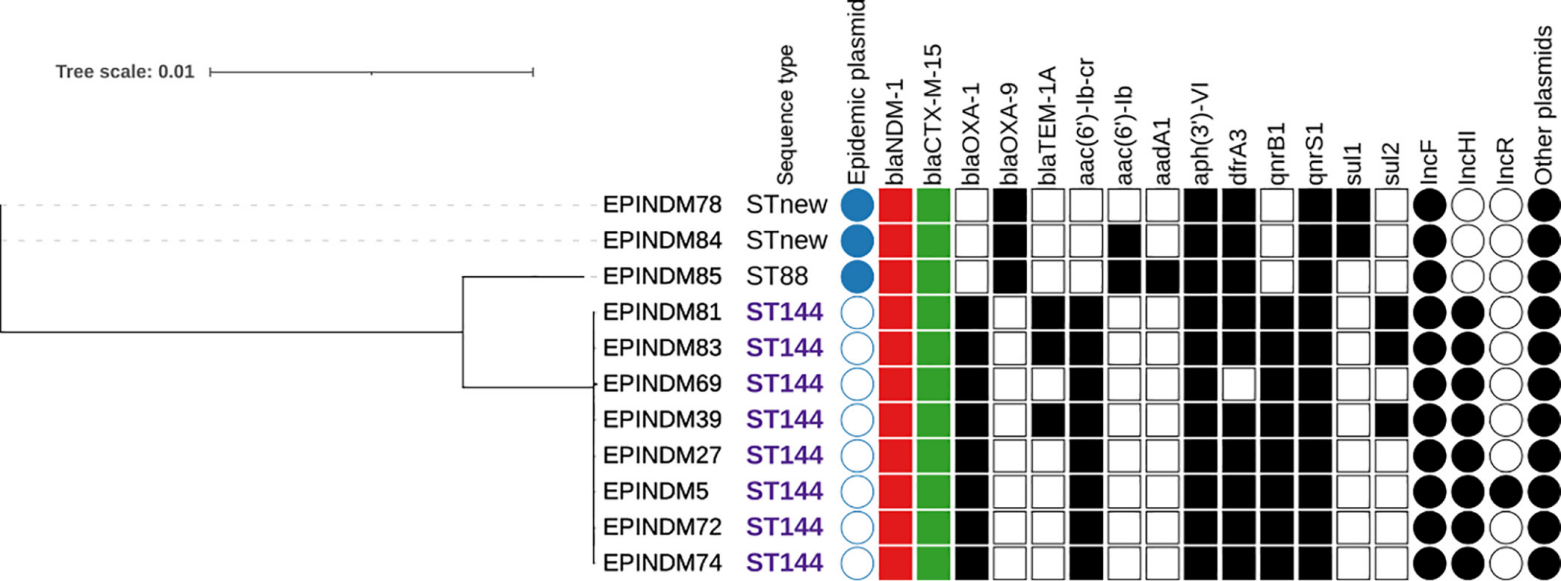
Klebsiella oxytoca phylogenetic tree based on single nucleotide polymorphisms. Filled shapes indicate the presence of the corresponding gene or plasmid.

A phylogenetic analysis was performed on the genomes of K. pneumoniae and K. oxytoca isolates, the predominant species (Fig. 3 and 4). The core genome of K. pneumoniae was composed of 2,321,883 bp or 2,649 genes. The phylogenetic analysis showed a high homology between the 43 ST307 K. pneumoniae isolates, since 40 (93%) differed only from zero to 18 single nucleotide polymorphisms (SNPs). Based on a recently proposed relatedness SNP threshold for the K. pneumoniae species, which is 18 SNPs (15), these isolates were considered clonal. Three ST307 isolates, EPINDM33, 73, and 76, displayed 20 to 91 SNPs compared to others (Fig. 3). Isolates EPINDM33 (27 to 91 SNPs) and EPINDM73 (13 to 77 SNPs) had low trimmed read counts, 0.7 million and 1.2 million, respectively, while the mean read count among all the sequenced genomes was 2.7 million reads. Low read counts were associated with lower-quality assemblies and could explain an increase in the SNP count. EPINDM76 (64 to 91 SNPs) had an average read coverage, but an insertion in the *mutS* gene was found and could induce hypermutation, thus the high SNP count.

The two ST36 K. pneumoniae isolates, isolated from patient P43, shared one SNP and were included because their resistance phenotype differed. ST147 isolates, which differed by 61 SNPs, were carried by P9 and P55, two distinct imported cases.

The core genome of the 11 K. oxytoca isolates contained 2,484,078 bp or 2,977 genes. All ST144 isolates differed from one to 18 SNPs (Fig. 4). Although a relatedness threshold is yet to be proposed for K. oxytoca, if we consider the SNP threshold of K. pneumoniae (15), the eight ST144 isolates can be considered clonal.

### Epidemic plasmid.

**FIG 5:**
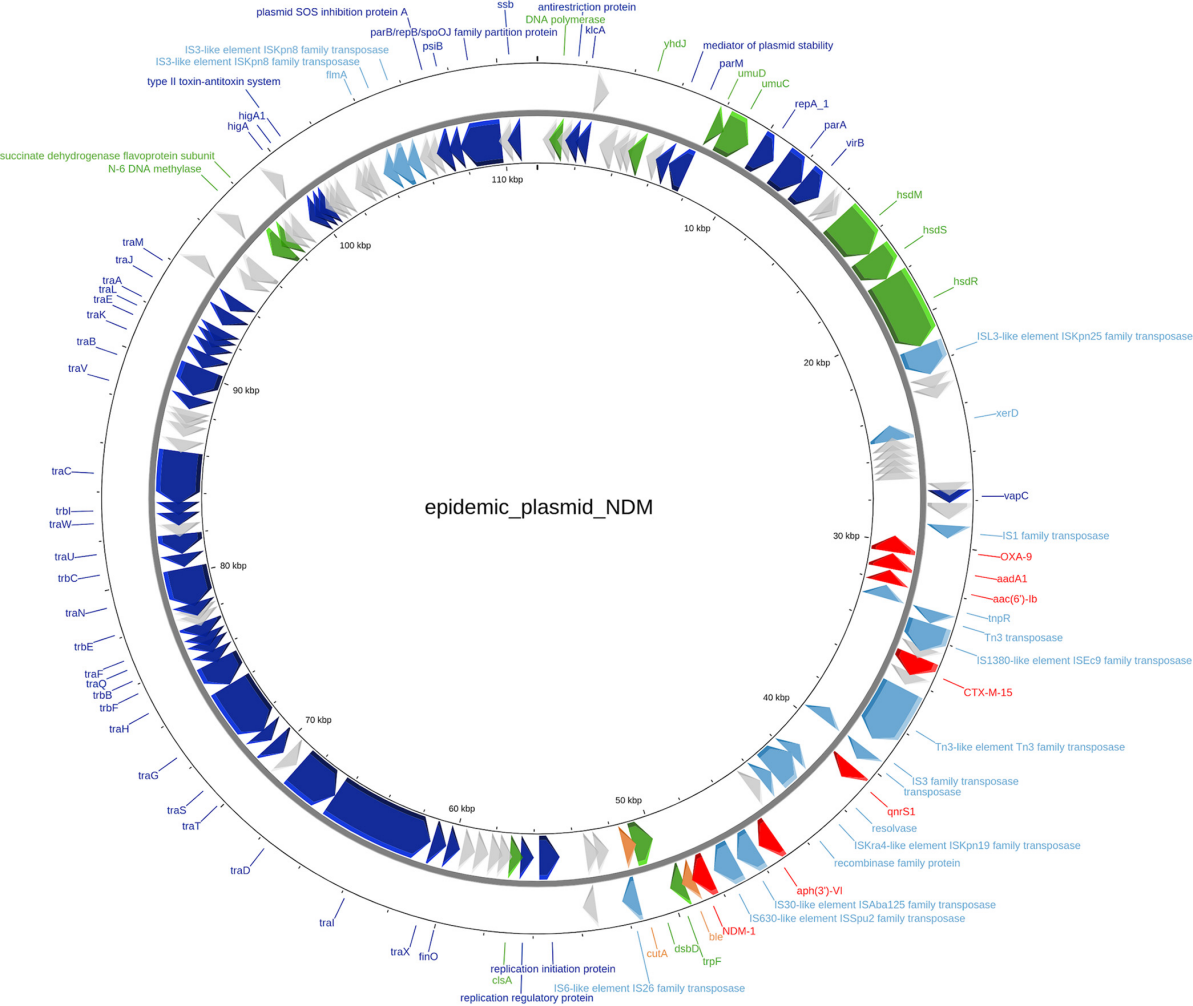
Annotated *bla*_NDM-1_-carrying epidemic plasmid. Red, antibiotic resistance genes. Orange, other resistance genes. Light blue, mobile genetic elements. Dark blue, conjugation genes. Green, maintenance genes. Gray, hypothetical genes.

The quality of the sequencing in the study provided a high number of trimmed reads per genome, 2.7 million on average, which allowed for the obtention of long contigs. In the genomes of epidemic isolates EPINDM24, 37, and 48, long plasmidic contigs carrying *bla*_NDM-1_ were retrieved and then assembled (Supplementary 6 figure), revealing a circularized plasmidic *bla*_NDM-1_-carrying sequence. The reconstructed plasmid had 110,000 bp and contained an IncFIIK [K2:A-/B-] replicon. In addition to the usual replication, conjugation, and maintenance genes, annotation successively identified seven resistance genes on this plasmid: *bla*_NDM-1_, *aph(3′)-VI*, *qnrS1*, *bla*_CTX-M-15_, *aac(6’)-Ib*, *aad1*, and *bla*_OXA-9_ (Fig. 5). Due to the limitations of Illumina short-read sequencing for plasmid reconstruction, Nanopore sequencing was performed on an ST307 K. pneumoniae isolate to confirm the presence and the characteristics of the epidemic plasmid. Nanopore sequencing showed the presence of a plasmid with 99.84% identity compared to the one reconstructed from the Illumina sequences.

The mapping of the 81 genomes on the reconstructed plasmid sequence showed that all 43 ST307 K. pneumoniae isolates and 18 of the 38 other isolates (47%) contained this plasmid, including 3/9 (33%) non-ST307 K. pneumoniae, 3/3 (100%) non-ST144 K. oxytoca, 8/11 (73%) E. coli, and 4/5 (80%) E. cloacae isolates. None of the ST144 K. oxytoca isolates harbored this plasmid. Eight patients simultaneously carried an ST307 K. pneumoniae isolates and an isolate of another species harboring the epidemic plasmid (Fig. 1 and Supplementary 1 table).

The sequence of this epidemic plasmid was compared to the National Center for Biotechnology Information database with the BLAST tool, and we found two results with >98% nucleotide similarity. One was from a hospital CPE outbreak in Germany (9) and the other from a K. pneumoniae ST395 isolate sequenced in an epidemiological surveillance of CPE in the United States (16).

### Genome analysis combined with epidemiologic data.

Genome analysis combined with patient data unraveled three dissemination phenomena running from August 2016: (i) a clonal dissemination of 43 K. pneumoniae ST307 strains; (ii) the spread of its *bla*_NDM-1_ plasmid to 18 other *Enterobacterales* isolates, affecting 44 patients; and (iii) a clonal dissemination of eight K. oxytoca ST144 isolates affecting seven patients (Fig. 1 and Supplementary 1 table). No patient was considered to be the “index case” because the first patient of the outbreak tested positive after 10 days of hospital stay. We therefore classified as acquired cases the 51 patients carrying at least one of the isolates of the dissemination phenomena. The 10 remaining were classified as imported cases.

Among the 38 cases initially considered acquired cases, 37 were also classified as such by WGS. Patient P19 actually carried an ST22 C. freundii isolate (EPINDM23) lacking the epidemic plasmid and was therefore reclassified as an imported case. Among the 10 cases initially classified as imported cases, nine were classified as such by WGS. Patient P10 was reclassified as an acquired case since the patient carried an E. cloacae isolate harboring the epidemic plasmid. Finally, the 13 cases epidemiologically classified as uncertain cases were all part of the outbreak including six cases of K. pneumoniae ST307, two cases of *Enterobacterales* carrying the epidemic plasmid, and 5 cases of K. oxytoca ST144. All were reclassified as acquired cases (Supplementary 1 table).

After the WGS reclassification of cases, epidemiological links could be established between all patients carrying K. pneumoniae ST307 and *Enterobacterales* harboring the epidemic plasmid. In contrast, no link, either spatially or temporally, was found to explain a cross-transmission of 5/7 K. oxytoca ST144 carriers, even after extensive investigation of hospital patient movements. Indeed, prolonged periods of up to 3 months without any NDM K. oxytoca-carrying patients were observed (Fig. 1).

## DISCUSSION

Through WGS, this NDM-CPE outbreak analysis revealed three simultaneous phenomena: the clonal dissemination of K. pneumoniae ST307; the spread of its *bla*_NDM-1_-carrying plasmid, which transferred to other *Enterobacterales* species; and the simultaneous clonal dissemination of ST144 K. oxytoca.

More than half of the NDM-CPE isolates isolated during the epidemic period were clonal ST307 K. pneumoniae. Carbapenemase-producing K. pneumoniae ST307 emerged globally. This ST has been described from hospital settings and associated with KPC-type carbapenemase in Italy (17–19), in South Africa (20), and in a South Korean hospital outbreak (10). Moreover, in Italy, ST307 is one of the most-represented STs among carbapenemase-producing K. pneumoniae isolates along with ST512 and ST258 (21). In Germany, a K. pneumoniae ST307 clonal outbreak was described in a hospital setting where the clone produced NDM-1 and OXA-48 and was shown to display virulence features including hypermucoviscosity, high siderophore secretion, and metabolite transporter capacities (20). In contrast, our ST307 K. pneumoniae clones did not feature any common virulence gene. Capsule typing showed that the ST307 K. pneumoniae isolates was associated with the KL102 capsular type, which has been described in severe infections (22). The CRACKLE-2 study (23) found that K. pneumoniae ST307 was the second most prevalent CPE (95% associated with *bla*_KPC-2_), after K. pneumoniae ST258, across 49 American hospitals between 2016 and 2017. K. pneumoniae ST307 was also described as highly associated with extended-spectrum β-lactamases (ESBL) in a study from Houston, TX, USA, in which ST307 was more prevalent than ST258 among 1,777 hospital ESBL-producing K. pneumoniae isolates (24). The global dissemination of this ST and its potential association with ESBL, carbapenemases, and virulence factors makes it an emerging “high-risk clone” (25).

The clonal K. pneumoniae ST307 was found to harbor an IncFIIK [K2:A-/B-] plasmid containing the *bla*_NDM-1_ gene but also six other resistance genes, *aph(3′)-VI*, *qnrS1*, *bla*_CTX-M-15_, *aac(6’)-Ib*, *aadA1*, and *bla*_OXA-9_, the combination of which confers a multidrug-resistant phenotype. This conjugative plasmid was observed in the genomes of several other *Enterobacterales* isolates of different species. Indeed, eight patients coharbored different species isolates which possessed the epidemic plasmid. This finding suggests interspecies transfers of the plasmid probably within the microbiota of the patients, since several patients were simultaneously carriers of an ST307 K. pneumoniae isolate and another *Enterobacterales* species harboring the epidemic plasmid. A previous study published in 2016 by Sheppard et al. used WGS on a collection of CPE isolates and demonstrated that in a North American hospital setting, *bla*_KPC_ emerged over time, not only via clonal spread but also on multiple genetic levels such as conjugation and transposition (26). This suggests that the investigation of CPE hospital outbreaks should not be limited to the search of disseminating clones and particularly when the outbreak lasts for years.

Surprisingly, we observed a simultaneous dissemination of clonal K. oxytoca ST144 isolates. We did not find any epidemiological link from the patients, since most of the K. oxytoca ST144 carriers did not share the same unit at the same time. A common source of contamination was investigated but remained undefined. Hypotheses including environmental contamination, ST144 carriers who were missed because they were not screened, and colonization of invasive procedure equipment were discussed but were difficult to test retrospectively. To our knowledge, the K. oxytoca species has not been reported as associated with a hospital outbreak, making our report the first of a carbapenemase-producing K. oxytoca ST144 hospital dissemination.

The WGS analysis disclosed the microbiological components and transmission routes involved in this epidemic, which could not have been achieved using classical epidemiological and microbiological phenotypic data alone. Older methods such as pulsed-field gel electrophoresis, repetitive element sequence-based PCR (Rep-PCR), or Sanger MLST sequencing might have been able to provide data on strain dissemination, although they have less discriminatory power than WGS, but they could not have identified the dissemination of the epidemic plasmid. Nevertheless, WGS results, as well as the number of SNPs suggested in the literature to confirm that two strains are similar, should not be interpreted roughly but should be coupled to the epidemiological analysis. Through its high resolution in genome comparison, WGS also allowed us to categorize uncertain cases, and to correct the classification of two other cases, demonstrating that epidemiological data analysis is not error proof. Nevertheless, even using this powerful typing method, CPE transmission cannot always be clarified with patient spatial-temporal data, e.g., the clonal spread of ST144 K. oxytoca in this work, which led to an unsolved investigation for a common environmental source. In line with previous publications (10, 27, 28), this retrospective study highlights the benefits of combining WGS with epidemiological data for outbreak analysis and raises the question of the impact of real-time WGS for epidemic surveillance and control. Systematic and real-time genomic characterization of newly detected CPE could rapidly confirm transmission between patients and speed up the implementation of appropriate control measures.

This study presents some nonoptimal points regarding CPE screening and sequencing methods. CPE surveillance in our hospital follows national recommendations, and screening is limited to patients with a history of CPE carriage or a recent hospital stay abroad and to contact patients. In the absence of exhaustive screening, some patients with CPE were probably missed, which could in part explain the duration of the outbreak. Moreover, the genomes of isolates with low read counts could not be resequenced, and our hypothesis linking the number of SNPs to the quality of sequencing remains uncertain. At least, we cannot exclude with certainty that there was no reintroduction of the epidemic plasmid into our hospital from the community or from other institutions, although this hypothesis seems unlikely because of the small number of similar plasmids found in the NCBI database, which contains more than 200,000 bacterial genomes.

### Conclusion.

Whole-genome analysis of all NDM-CPE isolated during our hospital outbreak unraveled its mechanisms, namely, the dissemination of a clonal NDM-producing ST307 K. pneumoniae isolate and its conjugative plasmid, but also that of an unexpected clonal spread of ST144 K. oxytoca whose origin remains to be determined.

## MATERIALS AND METHODS

### Setting.

We conducted a retrospective study at the 850-bed BCB hospital, which serves an adult population of more than 350,000 inhabitants and is located in an area with a large proportion of foreign-born patients originating primarily from Africa. It includes all medical and surgical specialties, except neurosurgery, with a heart, kidney, and lung transplant program. It has 3 intensive care units (ICUs), with a total of 61 beds, including a 26-bed medical ICU, an 18-bed surgical ICU, and a 17-bed cardiac surgical ICU.

### Control measures.

During the outbreak, and to this day, our hospital has followed the French national recommendations for controlling highly resistant bacteria, including glycopeptide-resistant enterococci and CPE (4). The recommendations require screening patients entering the hospital with a history of hospital stay abroad during the previous year and those previously known to be carrying highly resistant bacteria. Extensive screening is also performed for contact patients, defined as those sharing the same nursing staff as the case patient, and all patients hospitalized in the same unit as the case as well as contact patients already transferred to another unit or hospital at the time of CPE identification. Weekly screening is performed in wards as long as a case patient is present in the ward. Admission and transfer of patient in a ward with a fortuitous CPE detection are not allowed until contact patients have been screened at least twice. An automatic alert system allows identifying case and contact patients when readmitted to the hospital (29). Criteria for considering that a contact patient is not colonized or that a case patient is no longer a carrier are strict (4).

Contact precautions (CP) are implemented with the contact patient until suspicion is removed, preferentially with dedicated nursing staff. In situations with more than one case, cohorting with dedicated nursing staff, in a distinct location of the clinical ward, is implemented. Such a location of the infectious diseases department can accommodate case patients originating from different clinical wards.

### Definitions.

A case patient was defined as a patient having a positive culture with NDM-CPE, either from a screening sample or from a clinical culture, during the epidemic period, whatever the clinical unit of identification. Patients were classified as carriers if a rectal screening culture was positive alone during their hospital stay. If clinical cultures were positive, patients were classified as infected when the sampling was performed in a context of infection, according to standard definition (30), and as colonized when the sampling was performed without signs of infection.

NDM-CPE cases were classified independently from the WGS analysis as acquired, uncertain, or imported cases, based on epidemiologic evidence. Acquired cases were defined by the presence of another NDM-CPE case patient in the same ward during the stay of the newly acquired case, whatever the species of *Enterobacterales* carrying *bla*_NDM_. Imported cases were defined as patients with a positive culture of NDM-CPE within 48 h of hospital admission, who were not known as previous carriers and had no history of recent stay in our hospital. Other cases were classified as uncertain.

### Microbiological methods.

CPE screening was performed using a rectal swab (ESwab; Copan, CA, USA) collected by nursing staff or by the patients themselves. One hundred microliters of the e-Swab liquid (Amies medium) was inoculated in a buffered dextrose broth (Bio-Rad, CA, USA) supplemented with 0.5 mg/L of ertapenem and cultured overnight (18 to 24 h at 35 ± 2°C).

During a first period, July 2016 to February 2017, the enrichment broth was plated on Drigalski agar with an ertapenem and an imipenem Etest strip (bioMérieux, Marcy-l’Etoile, France) and incubated overnight (18 to 24 h at 35 ± 2°C) under aerobic conditions. All colonies growing in the ellipse of >0.5 mg/L of ertapenem or >2 mg/L of imipenem were identified (31). After February 2017, the enrichment broth was plated onto two chromogenic selective agar media, chromID CARBA and chromID OXA48 (bioMérieux, Marcy-l’Etoile, France), overnight (18 to 24 h at 35 ± 2°C) under aerobic conditions. All colonies growing on these media were identified using mass spectrometry (MALDI Biotyper; Bruker Daltonics, Bremen, Germany). For *Enterobacterales*, the antibiotic susceptibility was determined by the disc diffusion method and interpreted according to EUCAST (www.eucast.org). Carbapenemase genes were confirmed using the GeneXpert Carba-R assay (Cepheid, Sunnyvale, CA, USA) according to the manufacturer’s recommendations (32). All CPE isolates were stored at −80°C.

In situations in which immediate decisions were required with respect to implementing CP, cohorting CPE-positive patients, or transferring a contact patient, a GeneXpert Carba-R assay was also performed directly from the ESwab. These situations most frequently included admission to the hospital after a history of hospital stay abroad and first contact tracing around a patient newly identified as CPE infected or colonized during his/her hospital stay.

### Whole-genome sequencing.

The first NDM-CPE isolate per patient, including clinical and screening samples, was selected for WGS analysis, except in a case of different species or different antimicrobial susceptibility pattern, i.e., a change of categorization from susceptible to intermediary/resistant for an antibiotic or vice versa. Bacterial DNA extraction was performed using the EZ1 DNA tissue kit (Qiagen, Hilden, Germany) on the EZ1 Advanced XL device (Qiagen). DNA concentrations were measured with NanoDrop 2000c (Thermo Fisher Scientific, Waltham, MA, USA). DNA extracts were shipped to LGC Group (LGC Genomics, Berlin, Germany), for library preparation and sequencing. WGS was performed on a NextSeq platform, Illumina (San Diego, CA, USA) with a midoutput kit which provides paired-end reads of 150 bp and an output of 32.5 to 39 billion bases for an intended sequencing depth above 50×. An isolate containing the epidemic plasmid was sequenced using Nanopore technology to reconstruct the plasmid with long reads (33). Libraries were prepared using the rapid barcoding SQK-RBK004 kit (Oxford Nanopore Technologies, Oxford, UK). Whole-genome sequencing was performed with a Flongle flow cell (R9.4.1), on a MinION platform (Oxford Nanopore Technologies).

### Bioinformatic analysis.

Raw Illumina reads were trimmed and filtered using Trimmomatic v0.39 (34) and Trim Galore v0.5.0 (35). Filters used were Phred score >30 and read length >50 bases. Raw and trimmed read quality were assessed with FastQC v0.11.7 (36). Species identification and cross-contamination were checked with MetaPhlAn v2.0 (37). Genome *de novo* assemblies were performed with SPAdes v3.12 (38), and assembly quality was examined by QUAST v5 (39). Long read sequences were assembled using Flye 2.9 (40). Gene annotation was performed by Prokka v1.13 (41). Resistance genes and plasmid replicons were identified by Diamond v0.9.22 (42) with ResFinder (2019-02-20 version) and PlasmidFinder (2018-11-20 version) databases, respectively. Multilocus sequence typing (MLST) was achieved with the PubMLST.org database (43). The Kleborate pipeline was used to look for virulence genes in Klebsiella isolates (44). Polysaccharide capsule typing of K. pneumoniae was performed using Kaptive Web (https://kaptive-web.erc.monash.edu/) (45). Phylogeny analyses were performed using Parsnp v1.12 (46), MAFFT v7.407 (47), and PhyML v3.0 (48). K. pneumoniae genomes differing by 18 SNPs or fewer were considered similar (suggesting a high probability of transmission) (15). As no threshold is yet proposed for Klebsiella oxytoca, the same threshold as for K. pneumoniae was used.

### Ethics statement.

This study was conducted retrospectively, including only samples that were collected in the routine care of the patient. No additional samples were taken for this study, and no clinical data were collected. This study did not require any specific ethical approval as it used demographic data that were extracted anonymously according to current French regulations. All patients admitted to AP-HP hospitals are informed about the potential use of their data for research purposes.

### Data availability.

Raw Illumina sequence data are available from the National Center for Biotechnology Information under BioProject accession number PRJNA738476.
