# Supplementary material for: Simultaneous Hospital Outbreaks of New Delhi Metallo-β-Lactamase-Producing Enterobacterales Unraveled Using Whole-Genome Sequencing
Source: Microbiol Spectr. 2022 Mar 21;10(2):e02287-21. doi: 10.1128/spectrum.02287-21 (PMC9045244; doi:10.1128/spectrum.02287-21)

Supplementary 1: Overview table of sequenced isolates and their carriers.

Isolate	Patient	Sampling location	Origin	Sampling date	Species	ST	Epidemic plasmid	Outbreak analysis	Revised analysis
EPINDM1	1	Surgical intensive care	Rectal screening	09/08/2016	*K. pneumoniae*	307	Yes	Acquired case	Main outbreak
EPINDM2	1	Surgical intensive care	Urine	15/08/2016	*K. pneumoniae*	307	Yes	Acquired case	Main outbreak
EPINDM3	2	Surgical intensive care	Rectal screening	28/08/2016	*K. pneumoniae*	307	Yes	Uncertain case	Main outbreak
EPINDM4	3	Pneumology	Rectal screening	29/08/2016	*K. pneumoniae*	307	Yes	Uncertain case	Main outbreak
EPINDM5	4	Endocrinology	Rectal screening	30/08/2016	*K. oxytoca*	144	No	Uncertain case	Secondary outbreak
EPINDM6	5	Pneumology	Rectal screening	31/08/2016	*K. pneumoniae*	307	Yes	Acquired case	Main outbreak
EPINDM7	6	Emergency room	Rectal screening	01/09/2016	*E. cloacae*	171	No	Imported case	Imported case
EPINDM8	7	Infectiology	Rectal screening	07/09/2016	*K. pneumoniae*	307	Yes	Acquired case	Main outbreak
EPINDM10	8	Pneumology	Rectal screening	13/09/2016	*K. pneumoniae*	307	Yes	Acquired case	Main outbreak
EPINDM22	8	Pneumology	Rectal screening	12/12/2016	*K. pneumoniae*	161	Yes	Acquired case	Main outbreak
EPINDM40	8	Pneumology	Rectal screening	15/03/2017	*E. coli*	12	Yes	Acquired case	Main outbreak
EPINDM9	8	Pneumology	Rectal screening	13/09/2016	*E. cloacae*	114	Yes	Acquired case	Main outbreak
EPINDM11	9	Cardiology	Rectal screening	14/09/2016	*K. pneumoniae*	147	No	Imported case	Imported case
EPINDM12	10	Infectiology	Skin	19/09/2016	*E. cloacae*	114	Yes	Imported case	Main outbreak
EPINDM13	11	Cardiology	Rectal screening	15/10/2016	*K. pneumoniae*	307	Yes	Acquired case	Main outbreak
EPINDM14	12	Emergency room	Rectal screening	10/10/2016	*K. pneumoniae*	857	No	Imported case	Imported case
EPINDM15	13	Surgical intensive care	Rectal screening	18/10/2016	*K. pneumoniae*	307	Yes	Acquired case	Main outbreak
EPINDM16	14	Infectiology	Rectal screening	31/10/2016	*E. coli*	405	No	Imported case	Imported case
EPINDM17	15	Cardiology	Rectal screening	15/11/2016	*K. pneumoniae*	307	Yes	Acquired case	Main outbreak
EPINDM18	16	Cardiology	Rectal screening	15/11/2016	*K. pneumoniae*	307	Yes	Acquired case	Main outbreak
EPINDM19	17	Surgical intensive care	Throat screening	01/12/2016	*K. pneumoniae*	307	Yes	Acquired case	Main outbreak
EPINDM21	17	Surgical intensive care	Rectal screening	07/12/2016	*E. coli*	683	Yes	Acquired case	Main outbreak
EPINDM20	18	Surgical intensive care	Rectal screening	05/12/2016	*K. pneumoniae*	307	Yes	Uncertain case	Main outbreak
EPINDM23	19	Surgical intensive care	Rectal screening	26/12/2016	*C. freundii*	22	Yes	Acquired case	Imported case
EPINDM24	20	Surgical intensive care	Rectal screening	30/12/2016	*K. pneumoniae*	307	Yes	Acquired case	Main outbreak
EPINDM26	21	Cardiology	Rectal screening	08/01/2017	*K. pneumoniae*	307	Yes	Acquired case	Main outbreak
EPINDM27	22	Pneumology	Rectal screening	10/01/2017	*K. oxytoca*	144	No	Uncertain case	Secondary outbreak
EPINDM28	23	Digestive surgery	Rectal screening	16/01/2017	*K. pneumoniae*	307	Yes	Acquired case	Main outbreak
EPINDM41	23	Digestive surgery	Rectal screening	17/03/2017	*E. coli*	540	Yes	Acquired case	Main outbreak
EPINDM29	24	Infectiology	Blood culture	13/02/2017	*K. pneumoniae*	307	Yes	Acquired case	Main outbreak
EPINDM30	25	Surgical intensive care	Rectal screening	20/02/2017	*K. pneumoniae*	307	Yes	Acquired case	Main outbreak
EPINDM36	25	Surgical intensive care	Blood culture	24/02/2017	*K. pneumoniae*	307	Yes	Acquired case	Main outbreak
EPINDM31	26	Medical intensive care	Throat screening	20/02/2017	*K. pneumoniae*	307	Yes	Acquired case	Main outbreak
EPINDM37	26	Medical intensive care	Throat screening	28/02/2017	*E. coli*	416	Yes	Acquired case	Main outbreak
EPINDM32	27	Infectiology	Rectal screening	20/02/2017	*K. pneumoniae*	307	Yes	Acquired case	Main outbreak
EPINDM33	28	Surgical intensive care	Skin	22/02/2017	*K. pneumoniae*	307	Yes	Acquired case	Main outbreak
EPINDM34	29	Medical intensive care	Catheter	24/02/2017	*E. cloacae*	145	Yes	Acquired case	Main outbreak
EPINDM35	30	Infectiology	Rectal screening	23/02/2017	*K. pneumoniae*	307	Yes	Acquired case	Main outbreak
EPINDM38	31	Pneumology	Rectal screening	07/03/2017	*K. pneumoniae*	307	Yes	Acquired case	Main outbreak
EPINDM39	32	Medical intensive care	Rectal screening	12/03/2017	*K. oxytoca*	144	No	Uncertain case	Secondary outbreak
EPINDM42	33	Pneumology	Rectal screening	26/03/2017	*K. pneumoniae*	307	Yes	Acquired case	Main outbreak
EPINDM43	34	Surgical intensive care	Rectal screening	28/03/2017	*K. pneumoniae*	307	Yes	Acquired case	Main outbreak
EPINDM44	34	Surgical intensive care	Rectal screening	09/01/2018	*E. coli*	1851	Yes	Acquired case	Main outbreak
EPINDM52	34	Surgical intensive care	Rectal screening	09/05/2017	*E. coli*	1851	Yes	Acquired case	Main outbreak
EPINDM85	34	Surgical intensive care	Rectal screening	09/01/2018	*K. oxytoca*	88	Yes	Acquired case	Main outbreak
EPINDM46	35	Cardiology	Rectal screening	31/03/2017	*K. pneumoniae*	307	Yes	Acquired case	Main outbreak
EPINDM66	35	Cardiology	Rectal screening	26/07/2017	*E. coli*	20	Yes	Acquired case	Main outbreak
EPINDM47	36	Surgical intensive care	Rectal screening	31/03/2017	*K. pneumoniae*	307	Yes	Acquired case	Main outbreak
EPINDM48	37	Surgical intensive care	Rectal screening	01/04/2017	*E. coli*	131	Yes	Acquired case	Main outbreak
EPINDM49	37	Surgical intensive care	Rectal screening	01/04/2017	*K. pneumoniae*	307	Yes	Acquired case	Main outbreak
EPINDM50	38	Surgical intensive care	Rectal screening	10/04/2017	*K. pneumoniae*	307	Yes	Acquired case	Main outbreak
EPINDM54	38	Surgical intensive care	Rectal screening	16/06/2017	*K. pneumoniae*	307	Yes	Acquired case	Main outbreak
EPINDM51	39	Emergency room	Urine	10/04/2017	*K. pneumoniae*	15	Yes	Imported case	Imported case
EPINDM53	40	Pneumology	Rectal screening	16/05/2017	*K. pneumoniae*	307	Yes	Acquired case	Main outbreak
EPINDM55	41	Surgical intensive care	Throat screening	16/05/2017	*K. pneumoniae*	307	Yes	Acquired case	Main outbreak
EPINDM56	42	Surgical intensive care	Rectal screening	06/06/2017	*K. pneumoniae*	307	Yes	Acquired case	Main outbreak
EPINDM57	43	Surgical intensive care	Rectal screening	20/06/2017	*K. pneumoniae*	36	Yes	Acquired case	Main outbreak
EPINDM60	43	Surgical intensive care	Catheter	29/06/2017	*K. pneumoniae*	36	Yes	Acquired case	Main outbreak
EPINDM58	44	Surgical intensive care	Rectal screening	22/06/2017	*K. pneumoniae*	307	Yes	Acquired case	Main outbreak
EPINDM76	44	Surgical intensive care	Rectal screening	23/11/2017	*K. pneumoniae*	307	Yes	Acquired case	Main outbreak
EPINDM77	44	Surgical intensive care	Rectal screening	23/11/2017	*E. cloacae*	995	Yes	Acquired case	Main outbreak
EPINDM59	45	Emergency room	Rectal screening	26/06/2017	*E. coli*	361	No	Imported case	Imported case
EPINDM61	46	Infectiology	Rectal screening	01/07/2017	*C. freundii*	22	Yes	Uncertain case	Main outbreak
EPINDM71	46	Infectiology	Rectal screening	07/09/2017	*K. pneumoniae*	307	Yes	Uncertain case	Main outbreak
EPINDM62	47	Emergency room	Rectal screening	06/07/2017	*E. coli*	636	No	Imported case	Imported case
EPINDM63	47	Emergency room	Rectal screening	07/07/2017	*K. pneumoniae*	1998	No	Imported case	Imported case
EPINDM64	48	Infectiology	Rectal screening	19/07/2017	*K. pneumoniae*	307	Yes	Acquired case	Main outbreak
EPINDM65	49	Infectiology	Rectal screening	20/07/2017	*K. pneumoniae*	307	Yes	Acquired case	Main outbreak
EPINDM67	50	Surgical intensive care	Pulmonary	18/08/2017	*K. pneumoniae*	11	No	Imported case	Imported case
EPINDM69	51	Surgical intensive care	Rectal screening	24/08/2017	*K. oxytoca*	144	No	Acquired case	Secondary outbreak
EPINDM70	52	Infectiology	Rectal screening	31/08/2017	*K. pneumoniae*	307	Yes	Acquired case	Main outbreak
EPINDM72	53	Pneumology	Rectal screening	29/09/2017	*K. oxytoca*	144	No	Acquired case	Secondary outbreak
EPINDM74	53	Pneumology	Rectal screening	03/10/2017	*K. oxytoca*	144	No	Acquired case	Secondary outbreak
EPINDM73	54	Surgical intensive care	Rectal screening	03/10/2017	*K. pneumoniae*	307	Yes	Uncertain case	Main outbreak
EPINDM75	55	Infectiology	Rectal screening	10/11/2017	*K. pneumoniae*	147	No	Imported case	Imported case
EPINDM78	56	Nephrology	Rectal screening	08/12/2017	*K. oxytoca*	new	Yes	Uncertain case	Main outbreak
EPINDM80	57	Surgical intensive care	Rectal screening	19/12/2017	*K. pneumoniae*	307	Yes	Uncertain case	Main outbreak
EPINDM81	58	Dermatology	Rectal screening	26/12/2017	*K. oxytoca*	144	No	Uncertain case	Secondary outbreak
EPINDM82	59	Digestive surgery	Rectal screening	03/01/2018	*K. pneumoniae*	307	Yes	Acquired case	Main outbreak
EPINDM83	60	Medical intensive care	Rectal screening	24/01/2018	*K. oxytoca*	144	No	Uncertain case	Secondary outbreak
EPINDM84	61	Surgical intensive care	Rectal screening	11/01/2018	*K. oxytoca*	new	Yes	Uncertain case	Main outbreak

Supplementary 2: Species distribution of sequenced isolates.

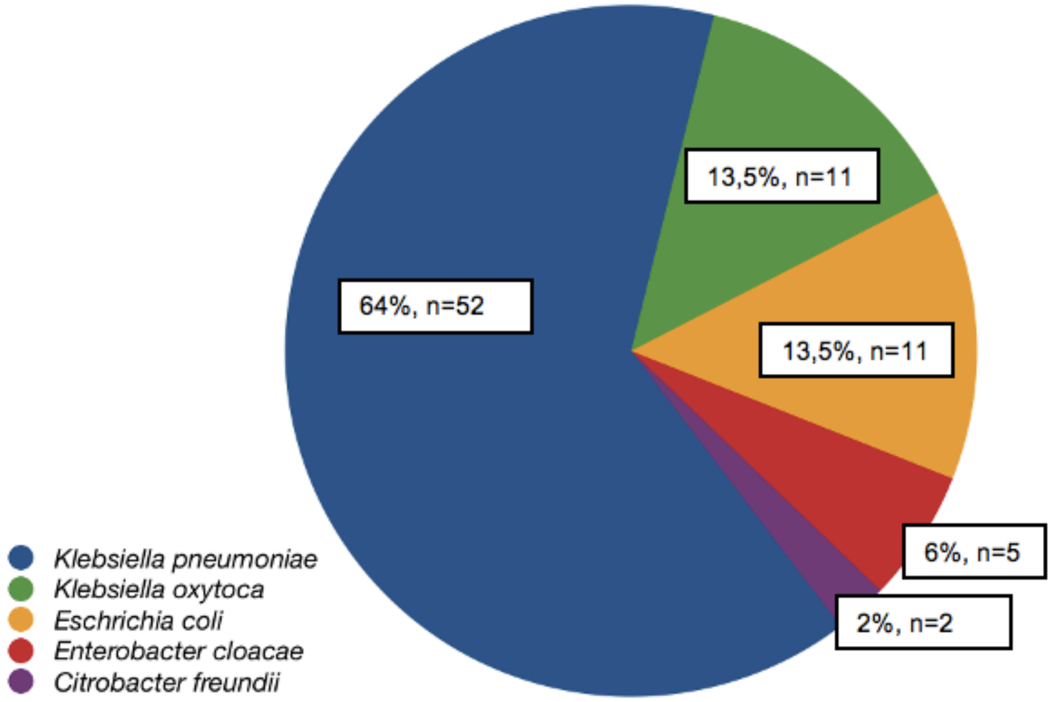

Supplementary 3: Phenotypic resistance of the sequenced isolates against antibiotics.

**Antibiotic**	**Resistant isolates (%)**
Amoxicillin	81 (100%)
Amoxicillin + clavulanic acid	81 (100%)
Ticarcillin	81 (100%)
Ticarcillin + clavulanic acid	81 (100%)
Piperacillin	81 (100%)
Piperacillin + tazobactam	81 (100%)
Cefoxitin	81 (100%)
Cefotaxime	81 (100%)
Ceftazidime	81 (100%)
Cefepime	81 (100%)
Imipenem	81 (100%)
Meropenem	81 (100%)
Ofloxacine	73 (90%)
Ciprofloxacine	73 (90%)
Gentamicin	25 (31%)
Amikacin	16 (20%)

Supplementary 4a: Resistance genes identified in the genomes of sequenced isolates using the ResFinder database.

Patient	Isolate	Species	ST	Epidemic plasmid	ARR-3_1_JF806499	ARR-6_1_FM955585	aac(3)-IIa_1_X51534	aac(3)-IId_1_EU022314	aac(6')-IIc_1_NC_012555	aac(6')-Ib-cr_1_DQ303918	aac(6')-Ib_1_M21682	aadA14_1_AJ884726	aadA16_1_EU675686	aadA1_4_JQ480156	aadA2_1_X68227	aadA5_1_AF137361	aph(3'')-Ib_2_AF024602	aph(3')-Ia_5_AP004237	aph(3')-VI_1_KC170992	aph(6)-Ic_1_X01702	aph(6)-Id_1_M28829	blaACC-1_2_HG530658	blaCMH-3_1_KX192165	blaCMY-4_1_LNHZ01000079	blaCTX-M-14_1_AF252622	blaCTX-M-15_1_AY044436	blaCTX-M-82_1_DQ256091	blaDHA-7_1_HQ456945	blaGES-1_1_HQ170511	blaLEN3_1_AY130286	blaNDM-1_1_FN396876	blaNDM-5_1_JN104597	blaNDM-7_1_JX262694	blaOKP-B-14_1_DQ995288	blaOXA-1_1_HQ170510	blaOXA-48_2_AY236073	blaOXA-9_1_KQ089875	blaTEM-12_1_M88143	blaTEM-1A_1_HM749966	blaTEM-2_1_X54606	blaTER-2_1_FJ263090	catA1_1_V00622	catA2_1_X53796	catB4_1_AF322577	cat_1_M11587	dfrA12_8_AM040708	dfrA14_1_KF921535	dfrA15_2_AF221900	dfrA17_1_FJ460238	dfrA19_1_EU855687	dfrA1_8_X00926	dfrA3_1_J03306	ere(A)_2_AF099140	erm(B)_1_JN899585	floR_2_AF118107	fosA5_1_EU195449	fosA_1_M85195	fosA_3_ACWO01000079	fosA_6_ACZD01000244	fosA_7_AEXB01000013	mcr-3.6_1_MF598076	mph(A)_1_D16251	qnrB1_1_DQ351241	qnrB4_1_DQ303921	qnrB6_1_EF520349	qnrB7_1_EU043311	qnrE1_1_KY073238	qnrS1_1_AB187515	rmtB_1_AB103506	sul1_2_U12338	sul2_2_AY034138	tet(34)_1_AB061440	tet(A)_4_AJ517790	tet(B)_2_AF326777	tet(C)_3_AF055345	tet(D)_1_AF467077
1	EPINDM1	*K. pneumoniae*	307	Yes							X			X			X		X		X					X					X						X															X							X									X			X	X				
1	EPINDM2	*K. pneumoniae*	307	Yes			X				X			X			X		X		X					X					X				X		X		X													X							X									X			X					
2	EPINDM3	*K. pneumoniae*	307	Yes							X			X			X		X		X					X					X						X															X							X									X			X	X				
3	EPINDM4	*K. pneumoniae*	307	Yes							X			X			X		X		X					X					X						X															X							X									X			X	X				
4	EPINDM5	*K. oxytoca*	144	No			X			X								X	X			X				X					X				X												X					X				X							X					X				X				
5	EPINDM6	*K. pneumoniae*	307	Yes							X			X			X		X		X					X					X						X															X							X									X			X	X				
6	EPINDM7	*E. cloacae*	171	No			X			X																X							X		X										X		X					X					X						X											X	X			
7	EPINDM8	*K. pneumoniae*	307	Yes							X			X			X		X		X					X					X						X															X							X									X			X	X				
8	EPINDM9	*E. cloacae*	114	Yes			X				X						X		X		X					X					X				X		X		X							X		X				X	X					X						X					X		X	X	X	X			
8	EPINDM10	*K. pneumoniae*	307	Yes							X			X			X		X		X					X					X						X															X							X									X			X	X				
9	EPINDM11	*K. pneumoniae*	147	No	X						X			X					X							X					X				X		X		X					X									X						X										X		X		X				
10	EPINDM12	*E. cloacae*	114	Yes			X										X		X		X					X					X				X		X		X							X		X				X	X					X						X					X		X	X	X	X			
11	EPINDM13	*K. pneumoniae*	307	Yes							X			X			X		X		X					X					X						X															X							X									X			X	X				
12	EPINDM14	*K. pneumoniae*	857	No	X			X		X							X		X		X			X							X				X				X					X									X			X				X							X				X	X	X			X	
13	EPINDM15	*K. pneumoniae*	307	Yes							X			X	X				X							X					X				X		X		X							X						X							X			X						X		X	X	X				
14	EPINDM16	*E. coli*	405	No						X					X											X						X			X				X							X						X		X								X							X	X		X		X		
15	EPINDM17	*K. pneumoniae*	307	Yes							X			X			X		X		X					X					X						X															X							X									X			X	X				
16	EPINDM18	*K. pneumoniae*	307	Yes							X			X			X		X		X					X					X						X															X							X									X			X	X				
17	EPINDM19	*K. pneumoniae*	307	Yes							X			X			X		X		X					X					X						X															X							X									X			X	X				
18	EPINDM20	*K. pneumoniae*	307	Yes							X			X			X		X		X					X					X						X															X							X									X			X	X				
17	EPINDM21	*E. coli*	683	Yes							X			X	X		X	X	X		X					X					X						X		X							X						X										X						X		X	X	X	X			
8	EPINDM22	*K. pneumoniae*	161	Yes	X			X		X			X				X		X		X					X					X								X													X							X						X			X		X	X	X				X
19	EPINDM23	*Cl freundii*	22	No	X		X			X		X		X					X	X					X	X					X				X					X				X							X	X										X					X	X	X	X	X	X				X
20	EPINDM24	*K. pneumoniae*	307	Yes							X			X					X							X					X				X		X															X							X									X				X				
21	EPINDM26	*K. pneumoniae*	307	Yes							X			X			X		X		X					X					X						X															X							X									X				X				
22	EPINDM27	*K. oxytoca*	144	No			X			X								X	X			X				X					X				X												X					X				X							X					X				X				
23	EPINDM28	*K. pneumoniae*	307	Yes										X	X		X		X		X					X					X				X		X										X						X							X			X	X					X		X	X	X	X			
24	EPINDM29	*K. pneumoniae*	307	Yes							X			X			X		X		X					X					X						X															X							X									X			X	X				
25	EPINDM30	*K. pneumoniae*	307	Yes			X							X			X		X		X					X					X				X		X		X													X							X									X			X	X				
26	EPINDM31	*K. pneumoniae*	307	Yes							X			X			X		X		X					X					X						X															X							X									X			X	X				
27	EPINDM32	*K. pneumoniae*	307	Yes							X			X			X		X		X					X					X						X															X							X									X			X	X				
28	EPINDM33	*K. pneumoniae*	307	Yes							X			X			X		X		X					X					X						X															X							X									X			X	X				
29	EPINDM34	*E. cloacae*	145	Yes		X	X		X					X			X		X		X					X		X			X						X		X				X		X					X		X	X							X	X			X				X		X		X				
30	EPINDM35	*K. pneumoniae*	307	Yes							X			X			X		X		X					X					X						X															X							X									X			X	X				
25	EPINDM36	*K. pneumoniae*	307	Yes							X			X			X		X		X					X					X						X															X							X									X			X	X				
26	EPINDM37	*E. coli*	416	Yes							X			X					X							X					X						X															X																X				X				
31	EPINDM38	*K. pneumoniae*	307	Yes							X			X			X		X		X					X					X						X															X							X									X			X	X				
32	EPINDM39	*K. oxytoca*	144	No			X			X							X	X	X		X	X				X					X				X				X								X					X				X							X					X			X	X				
8	EPINDM40	*E. coli*	12	Yes							X			X			X		X		X					X					X						X										X					X																X			X	X				
23	EPINDM41	*E. coli*	540	Yes							X			X		X			X							X					X						X		X										X			X																X				X	X			

Supplementary 4b: Resistance genes identified in the genomes of sequenced isolates using the ResFinder database.

Patient	Isolate	Species	ST	Epidemic plasmid	ARR-3_1_JF806499	ARR-6_1_FM955585	aac(3)-IIa_1_X51534	aac(3)-IId_1_EU022314	aac(6')-IIc_1_NC_012555	aac(6')-Ib-cr_1_DQ303918	aac(6')-Ib_1_M21682	aadA14_1_AJ884726	aadA16_1_EU675686	aadA1_4_JQ480156	aadA2_1_X68227	aadA5_1_AF137361	aph(3'')-Ib_2_AF024602	aph(3')-Ia_5_AP004237	aph(3')-VI_1_KC170992	aph(6)-Ic_1_X01702	aph(6)-Id_1_M28829	blaACC-1_2_HG530658	blaCMH-3_1_KX192165	blaCMY-4_1_LNHZ01000079	blaCTX-M-14_1_AF252622	blaCTX-M-15_1_AY044436	blaCTX-M-82_1_DQ256091	blaDHA-7_1_HQ456945	blaGES-1_1_HQ170511	blaLEN3_1_AY130286	blaNDM-1_1_FN396876	blaNDM-5_1_JN104597	blaNDM-7_1_JX262694	blaOKP-B-14_1_DQ995288	blaOXA-1_1_HQ170510	blaOXA-48_2_AY236073	blaOXA-9_1_KQ089875	blaTEM-12_1_M88143	blaTEM-1A_1_HM749966	blaTEM-2_1_X54606	blaTER-2_1_FJ263090	catA1_1_V00622	catA2_1_X53796	catB4_1_AF322577	cat_1_M11587	dfrA12_8_AM040708	dfrA14_1_KF921535	dfrA15_2_AF221900	dfrA17_1_FJ460238	dfrA19_1_EU855687	dfrA1_8_X00926	dfrA3_1_J03306	ere(A)_2_AF099140	erm(B)_1_JN899585	floR_2_AF118107	fosA5_1_EU195449	fosA_1_M85195	fosA_3_ACWO01000079	fosA_6_ACZD01000244	fosA_7_AEXB01000013	mcr-3.6_1_MF598076	mph(A)_1_D16251	qnrB1_1_DQ351241	qnrB4_1_DQ303921	qnrB6_1_EF520349	qnrB7_1_EU043311	qnrE1_1_KY073238	qnrS1_1_AB187515	rmtB_1_AB103506	sul1_2_U12338	sul2_2_AY034138	tet(34)_1_AB061440	tet(A)_4_AJ517790	tet(B)_2_AF326777	tet(C)_3_AF055345	tet(D)_1_AF467077
33	EPINDM42	*K. pneumoniae*	307	Yes							X			X			X		X		X					X					X						X															X							X									X			X	X				
34	EPINDM43	*K. pneumoniae*	307	Yes							X			X			X		X		X					X					X						X															X							X									X			X	X				
34	EPINDM44	*E. coli*	1851	Yes							X			X					X							X					X						X															X																	X				X				
35	EPINDM46	*K. pneumoniae*	307	Yes							X			X			X		X		X					X					X						X															X							X									X			X	X				
36	EPINDM47	*K. pneumoniae*	307	Yes							X			X			X		X		X					X					X						X															X							X									X			X	X				
37	EPINDM48	*E. coli*	131	Yes							X			X					X							X					X						X				X											X																	X				X				
37	EPINDM49	*K. pneumoniae*	307	Yes							X			X			X		X		X					X					X						X															X							X									X			X	X				
38	EPINDM50	*K. pneumoniae*	307	Yes							X			X			X		X		X					X					X						X															X							X									X			X	X				
39	EPINDM51	*K. pneumoniae*	15	No			X			X				X	X			X								X				X	X				X				X							X						X							X			X	X							X		X				X
34	EPINDM52	*E. coli*	1851	Yes							X			X					X							X					X						X															X																	X				X				
40	EPINDM53	*K. pneumoniae*	307	Yes							X			X			X		X		X					X					X						X															X							X									X			X	X				
38	EPINDM54	*K. pneumoniae*	307	Yes							X			X			X		X		X					X					X						X															X							X									X			X	X				
41	EPINDM55	*K. pneumoniae*	307	Yes			X				X			X			X		X		X					X					X				X		X		X													X							X									X			X	X				
42	EPINDM56	*K. pneumoniae*	307	Yes							X			X			X		X		X					X					X						X															X							X									X			X	X				
43	EPINDM57	*K. pneumoniae*	36	Yes							X			X					X							X					X						X															X				X													X				X				
44	EPINDM58	*K. pneumoniae*	307	Yes							X			X			X		X		X					X					X						X															X							X									X			X	X				
45	EPINDM59	*E. coli*	361	No											X																	X													X						X										X								X		X	X			
43	EPINDM60	*K. pneumoniae*	36	Yes							X			X					X							X					X						X															X				X													X				X				
46	EPINDM61	*Cl freundii*	22	No	X					X		X		X					X	X						X					X				X			X						X							X	X										X					X	X	X	X	X	X				X
47	EPINDM62	*E. coli*	636	No				X						X	X		X				X					X						X							X			X				X					X	X										X								X	X	X		X		
47	EPINDM63	*K. pneumoniae*	1998	No			X			X					X											X						X		X	X				X				X			X						X							X			X						X		X	X	X				
48	EPINDM64	*K. pneumoniae*	307	Yes										X			X		X		X					X					X				X		X															X							X									X			X	X				
49	EPINDM65	*K. pneumoniae*	307	Yes							X			X			X		X		X					X					X						X															X							X									X			X	X				
35	EPINDM66	*E. coli*	20	Yes							X			X					X							X					X						X															X																	X				X				
50	EPINDM67	*K. pneumoniae*	11	No			X				X						X				X					X					X				X				X													X							X												X	X				
51	EPINDM69	*K. oxytoca*	144	No						X									X							X					X				X												X																X					X								
52	EPINDM70	*K. pneumoniae*	307	Yes							X			X			X		X		X					X					X						X															X							X									X			X	X				
46	EPINDM71	*K. pneumoniae*	307	Yes							X			X			X		X		X					X					X						X															X							X									X			X	X				
53	EPINDM72	*K. oxytoca*	144	No			X			X								X	X			X				X					X				X												X					X				X							X					X				X				
54	EPINDM73	*K. pneumoniae*	307	Yes							X			X			X		X		X					X					X						X															X							X									X			X	X				
53	EPINDM74	*K. oxytoca*	144	No			X			X								X	X			X				X					X				X												X					X				X							X					X				X				
55	EPINDM75	*K. pneumoniae*	147	importé			X			X							X				X						X				X				X	X			X													X						X					X							X		X	X			
44	EPINDM76	*K. pneumoniae*	307	Yes							X			X					X							X					X						X															X							X									X				X				
44	EPINDM77	*E. cloacae*	995	Yes															X				X			X					X																				X								X								X				X				
56	EPINDM78	*K. oxytoca*	new	Yes															X							X			X		X						X											X				X				X												X		X		X				
57	EPINDM80	*K. pneumoniae*	307	Yes							X			X			X		X		X					X					X						X															X							X									X			X	X				
58	EPINDM81	*K. oxytoca*	144	No			X			X							X	X	X		X	X				X					X				X				X								X					X				X							X					X			X	X				
59	EPINDM82	*K. pneumoniae*	307	Yes							X			X			X		X		X					X					X						X															X							X									X			X	X				
60	EPINDM83	*K. oxytoca*	144	No			X			X							X	X	X		X	X				X					X				X				X								X					X				X							X					X			X	X				
61	EPINDM84	*K. oxytoca*	new	Yes							X								X							X			X		X						X											X				X				X												X		X		X				
34	EPINDM85	*K. oxytoca*	88	Yes							X			X				X	X							X					X						X															X				X													X				X				

Supplementary 5: Plasmid incompatibility groups identified in the genomes of sequenced isolates, using the PlasmidFinder database.

Souche	MLST	Organisme	ColRNAI	IncA/C2	IncF	IncHI	IncI1	IncL/M	IncQ1	IncR	IncX3	IncY	TrfA
**EPINDM1**	307	*K pneumoniae*	X		X								
**EPINDM2**	307	*K pneumoniae*	X		X	X							
**EPINDM3**	307	*K pneumoniae*	X		X								
**EPINDM4**	307	*K pneumoniae*	X		X								
**EPINDM5**	144	*K oxytoca*	X		X	X				X			
**EPINDM6**	307	*K pneumoniae*	X		X		X						
**EPINDM7**	171	*E cloacae*	X								X		
**EPINDM8**	307	*K pneumoniae*	X		X								
**EPINDM9**	114	*E cloacae*	X		X	X							X
**EPINDM10**	307	*K pneumoniae*	X		X								
**EPINDM11**	147	*K pneumoniae*	X		X					X			
**EPINDM12**	114	*E cloacae*	X		X	X							X
**EPINDM13**	307	*K pneumoniae*	X		X								
**EPINDM14**	857	*K pneumoniae*	X	X	X								
**EPINDM15**	307	*K pneumoniae*	X		X								
**EPINDM16**	405	*E coli*	X		X								
**EPINDM17**	307	*K pneumoniae*	X		X								
**EPINDM18**	307	*K pneumoniae*	X		X								
**EPINDM19**	307	*K pneumoniae*	X		X								
**EPINDM20**	307	*K pneumoniae*	X		X								
**EPINDM21**	683	*E coli*	X		X		X						
**EPINDM22**	161	*K pneumoniae*	X		X					X			
**EPINDM23**	22	*Cl freundii*	X		X								
**EPINDM24**	307	*K pneumoniae*	X		X								
**EPINDM26**	307	*K pneumoniae*	X		X								
**EPINDM27**	144	*K oxytoca*	X		X	X							
**EPINDM28**	307	*K pneumoniae*	X		X								
**EPINDM29**	307	*K pneumoniae*	X		X								
**EPINDM30**	307	*K pneumoniae*	X		X	X							
**EPINDM31**	307	*K pneumoniae*	X		X								
**EPINDM32**	307	*K pneumoniae*	X		X								
**EPINDM33**	307	*K pneumoniae*	X		X								
**EPINDM34**	145	*E cloacae*	X		X	X							X
**EPINDM35**	307	*K pneumoniae*	X		X								
**EPINDM36**	307	*K pneumoniae*	X		X								
**EPINDM37**	416	*E coli*	X		X								
**EPINDM38**	307	*K pneumoniae*	X		X								
**EPINDM39**	144	*K oxytoca*	X		X	X							
**EPINDM40**	12	*E coli*	X		X								
**EPINDM41**	540	*E coli*	X		X		X						
**EPINDM42**	307	*K pneumoniae*	X		X								
**EPINDM43**	307	*K pneumoniae*	X		X								
**EPINDM44**	1851	*E coli*	X		X		X						
**EPINDM46**	307	*K pneumoniae*	X		X								
**EPINDM47**	307	*K pneumoniae*	X		X								
**EPINDM48**	131	*E coli*	X		X		X					X	
**EPINDM49**	307	*K pneumoniae*	X		X								
**EPINDM50**	307	*K pneumoniae*	X		X								
**EPINDM51**	15	*K pneumoniae*	X		X								
**EPINDM52**	1851	*E coli*	X		X								
**EPINDM53**	307	*K pneumoniae*	X		X								
**EPINDM54**	307	*K pneumoniae*	X		X								
**EPINDM55**	307	*K pneumoniae*	X		X	X							
**EPINDM56**	307	*K pneumoniae*	X		X								
**EPINDM57**	36	*K pneumoniae*	X		X								
**EPINDM58**	307	*K pneumoniae*	X		X								
**EPINDM59**	361	*E coli*	X		X							X	
**EPINDM60**	36	*K pneumoniae*	X		X								
**EPINDM61**	22	*Cl freundii*	X		X								
**EPINDM62**	636	*E coli*	X		X				X		X		
**EPINDM63**	1998	*K pneumoniae*	X		X	X				X	X		
**EPINDM64**	307	*K pneumoniae*	X		X	X							
**EPINDM65**	307	*K pneumoniae*	X		X								
**EPINDM66**	20	*E coli*	X		X								
**EPINDM67**	11	*K pneumoniae*	X		X					X			
**EPINDM69**	144	*K oxytoca*	X		X	X							
**EPINDM70**	307	*K pneumoniae*	X		X								
**EPINDM71**	307	*K pneumoniae*	X		X								
**EPINDM72**	144	*K oxytoca*	X		X	X							
**EPINDM73**	307	*K pneumoniae*	X		X	X							
**EPINDM74**	144	*K oxytoca*	X		X	X							
**EPINDM75**	147	*K pneumoniae*	X		X	X		X		X			
**EPINDM76**	307	*K pneumoniae*	X		X								
**EPINDM77**	995	*E cloacae*	X		X								
**EPINDM78**	new	*K oxytoca*	X		X								
**EPINDM80**	307	*K pneumoniae*	X		X								
**EPINDM81**	144	*K oxytoca*	X		X	X							
**EPINDM82**	307	*K pneumoniae*	X		X								
**EPINDM83**	144	*K oxytoca*	X		X	X							
**EPINDM84**	new	*K oxytoca*	X		X								
**EPINDM85**	88	*K oxytoca*	X		X								

Supplementary 6: Epidemic plasmid reconstruction. The black, blue and orange colored contigs belong to the genomes of EPINDM37, 48 and 24, respectively.

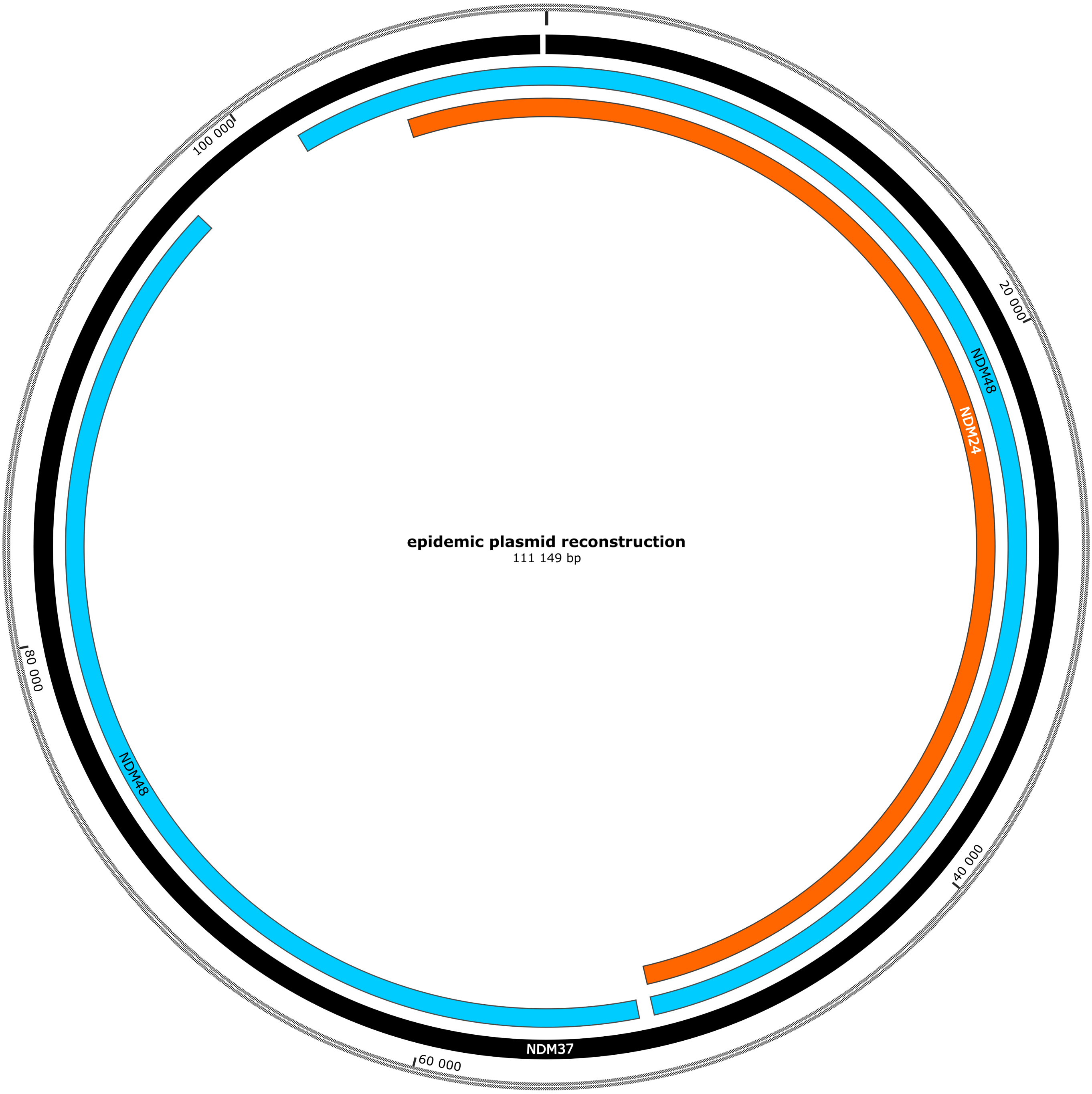

Supplement: SUPPLEMENTAL FILE 1 — Supplemental material. Download SPECTRUM02287-21_Supp_1_seq1.pdf, PDF file, 1.4 MB [file spectrum02287-21_supp_1_seq1.pdf]
